# Ratiometric Sensing of Hydrogen Peroxide Utilizing Conformational Change in Fluorescent Boronic Acid Polymers

**DOI:** 10.1155/2017/7829438

**Published:** 2017-09-28

**Authors:** Kan Takeshima, Kanako Mizuno, Hitoshi Nakahashi, Hiroshi Aoki, Yasumasa Kanekiyo

**Affiliations:** ^1^Department of Biotechnology and Environmental Chemistry, Kitami Institute of Technology, 165 Koen-cho, Kitami, Hokkaido 090-8507, Japan; ^2^National Institute of Advanced Industrial Science and Technology (AIST), 16-1 Onogawa, Tsukuba, Ibaraki 305-8569, Japan

## Abstract

We demonstrate that the copolymers containing boronic acid and pyrene units can be utilized for the fluorometric sensing of hydrogen peroxide (H_2_O_2_) in aqueous solutions. The copolymer exists in a relatively extended conformation in the absence of H_2_O_2_, whereas the polymer chain is contracted by the reaction of boronic acid moieties with H_2_O_2_ to form phenol groups. This conformational change induces aggregation of the originally isolated pyrene groups. As a result, relative intensity of excimer emission with respect to monomer emission increases with H_2_O_2_ concentration. Accordingly, the present methodology enables us to measure H_2_O_2_ by means of ratiometric fluorescence change in the range of 0–30 *μ*M.

## 1. Introduction

**Scheme 1:**
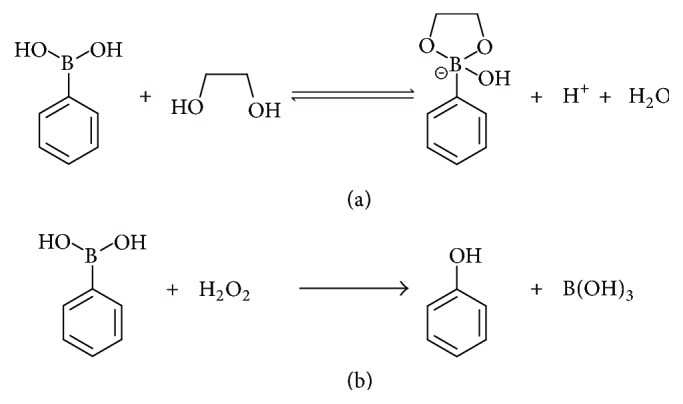
Reaction of boronic acid with saccharide (a) and hydrogen peroxide (b).

Boronic acids are known to form boronate esters with saccharides through reversible boronic acid-diol interactions (Scheme 1(a)) [1, 2]. By utilizing this nature, a variety of saccharide-sensing systems have been developed [3–5]. Boronic acids are also known to react with reactive oxygen species (ROS) such as hydrogen peroxide (H_2_O_2_) to generate the corresponding phenol derivatives (Scheme 1(b)) [6–12]. This reaction has been utilized for creating colorimetric [13–15] and fluorescent [16–20] probes for sensing H_2_O_2_ by directly connecting boronic acid moiety with chromophoric or fluorophoric compounds. When these probes react with H_2_O_2_, spectrophotometric changes are induced due to the alteration of the electronic state within the probes caused by the conversion of boronic acid moiety into phenol group. The boronic acid-based H_2_O_2_ probes are advantageous over the conventional enzyme-based sensors [21] in terms of durability and reproducibility because they do not use unstable bioorganic substances. One of the drawbacks of the boronic acid-based method may be the necessity of complicated organic synthesis for obtaining the probes.

Our group has been developing saccharide-sensing systems utilizing polymeric boronic acids. Previously, we had reported a novel saccharide detection strategy utilizing saccharide-induced conformational changes in fluorescent boronic acid polymers, which was synthesized from a radical copolymerization of a boronic acid monomer and a pyrene monomer [22]. This polymer takes a relatively contracted conformation in an aqueous solution due to hydrophobic aggregation of pyrene groups. On binding with saccharides, the boronic acid moieties become negatively charged, and the polymer chain changes its conformation to an extended state due to intramolecular electrostatic repulsion between the boronate groups. Thus, the pyrene groups are enforced to be dissociated. These conformational changes can be conveniently detected by monitoring the excimer to monomer intensity ratio in the fluorescence spectra. It occurred to us that a similar mechanism may be applied for developing sensing systems for H_2_O_2_. If the charge state of the boronic acid groups in the fluorescent polymer is altered by the reaction with H_2_O_2_, the conformation of the polymer chain will also be varied due to change in electrostatic interaction within the polymer. Thus, we expect that the concentration of H_2_O_2_ can be fluorometrically monitored using boronic acid polymers.

Here, we report our result on the endeavor for novel fluorometric sensing method for H_2_O_2_. Accordingly, we prepared copolymers containing boronic acid, acrylamide, and pyrene units with different compositions. After the reaction of the polymers with H_2_O_2_ in aqueous solutions, fluorescence spectral changes of the solutions were measured. As a result, we found that the relative intensity of excimer emission with respect to monomer emission increases with H_2_O_2_ concentration, and the present methodology enables us to measure H_2_O_2_ by means of ratiometric fluorescence change in the range of 0–30 *μ*M.

## 2. Experimental

### 2.1. Materials and Reagents

Boronic acid monomer** (1)** was synthesized according to the literature procedure [23] by the reaction between 3-aminophenylboronic acid and acryloyl chloride. Acrylamide** (2)** was purchased from Wako Pure Chemical Industries (Osaka, Japan). Pyrene monomer** (3)** was synthesized by a similar procedure described in the literature [24] by reacting 1-aminomethylpyrene and acryloyl chloride. Aqueous hydrogen peroxide (30 wt%) was purchased from Wako. Reagents for preparing buffer solutions [N-cyclohexyl-2-aminoethanesulfonic acid (CHES) and N-cyclohexyl-3-aminopropanesulfonic acid (CAPS)] were supplied from Dojindo Laboratories (Kumamoto, Japan).

### 2.2. Synthesis of Polymers

**Scheme 2:**
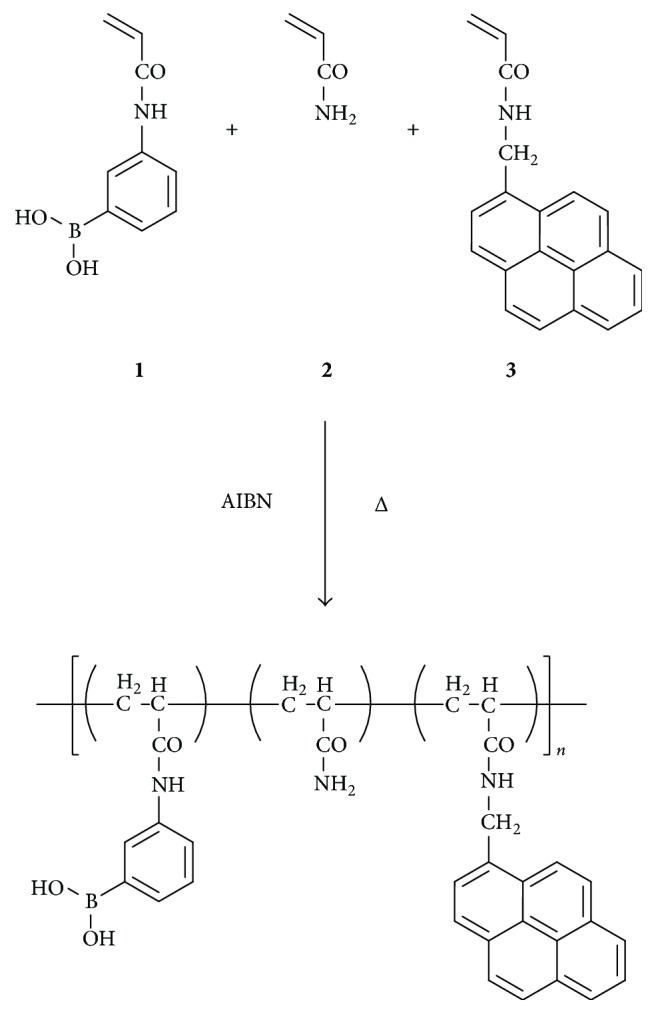
Preparation of fluorescent boronic acid polymers.

The outline for the preparation of the fluorescent boronic acid polymers is illustrated in Scheme 2. The radical copolymerization of** 1**,** 2,** and** 3** was carried out in homogeneous solutions in DMSO/H_2_O mixed solvent in the presence of a polymerization initiator (AIBN) at 70°C for 12 h under nitrogen atmosphere. For the monomer compositions, see Table 1. The obtained copolymers were purified by reprecipitation using acetone as a nonsolvent and then dried under vacuum. NMR spectra of the copolymers are shown in Figures S1–S5 (see Supplementary Material available online at https://doi.org/10.1155/2017/7829438). From these spectra, successful introduction of boronic acid and pyrene units was ascertained. Compositions of monomer units in the copolymers were estimated by elemental analysis. The observed C/N ratios approximately agreed with the calculated values as shown in Table S1. By using these data, molar ratios of monomer units were estimated (Table S2). It was confirmed that the molar ratios were roughly agreed with compositions in the feed solutions. Molecular weights (Mw) of these copolymers are supposed to be in the range of 13,000–22,000 [22].

### 2.3. Measurement of Fluorescence Spectra

The fluorescent boronic acid polymer was dissolved in methanol to be the polymer concentration of 0.1 g L^−1^. An aqueous solution containing H_2_O_2_ (0–100 *μ*M) and buffer (10 mM) was prepared and 8 mL of this solution was poured into a screw-capped bottle. To this solution was added 80 *μ*L of the polymer solution and stirred at 25°C for 60 min. After that, the fluorescence emission spectra of the solution were recorded using an excitation light at 348 nm. Other reagents were purchased from Wako Pure Chemical Industries (Osaka, Japan). The following buffers were used for setting pH of the solutions: 10 mM HEPES (pH 7.4), 10 mM CHES (pH 9.3–10.1), and 10 mM CAPS (pH 10.5–11.3).

### 2.4. Apparatus

Steady-state fluorescence spectra were recorded on Hitachi F-2500 fluorescence spectrophotometer. UV absorption spectra were obtained by using JASCO V-650 spectrophotometer. pH values were measured using Metrohm 827 pH lab. Aqueous solutions were prepared with distilled water purified by Yamato WG202 system.

## 3. Results and Discussion

### 3.1. Fluorometric Response against H_2_O_2_

**Figure 1:**
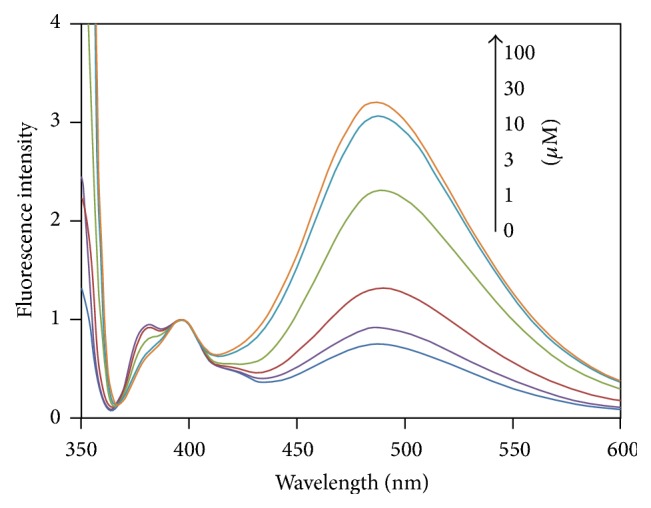
Fluorescence spectra for aqueous solutions of polymer B-11 (1 mg L^−1^) buffered at pH 10.9 by 10 mM CAPS at various H_2_O_2_ concentrations at 25°C. The spectra are normalized at 397 nm. Excitation: 348 nm.

The fluorescence spectroscopic characteristics of the synthesized copolymers were investigated in aqueous buffered solutions containing various concentrations of H_2_O_2_. The obtained fluoresce spectra are shown in Figures S6–S15. In the absence of H_2_O_2_, polymer B-11 having a 1 : 1 molar ratio between the boronic acid and acrylamide units showed a broad excimer emission in the range of 430–600 nm in addition to a monomer emission in the range of 370–410 nm (Figure 1). The excimer emission is derived from the dimerization between excited-state and ground state pyrene groups. The excitation spectra were also measured (Figure S16) and it was found that the spectrum monitored at the excimer emission wavelength (486 nm) was remarkably different from that monitored at monomer emission wavelength (397 nm). The observed excitation spectra indicate that the pyrene groups form aggregations in the ground state probably due to hydrophobic and *π*-*π* stacking interactions [25].

**Figure 2:**
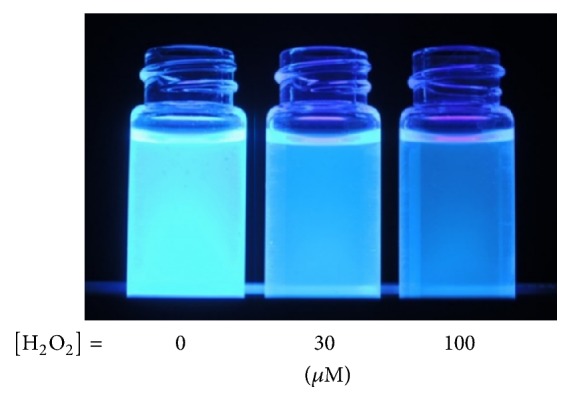
Visible change of the emission color from the aqueous solutions of polymer B-11 (10 mg L^−1^) containing or not containing H_2_O_2_.

When 1 *μ*M of H_2_O_2_ was added to the solution, the excimer emission was slightly intensified relative to the monomer emission. With increasing H_2_O_2_ concentration, the relative excimer intensity gradually increased and almost saturated above 30 *μ*M as shown in Figure 1. This spectral change accompanied a clearly visible variation in the emission color from the solution as shown in Figure 2. By measuring the corresponding UV absorption spectra (Figure S17), it was found that a new absorption assignable to phenol moiety emerged at around 290 nm with increasing H_2_O_2_ concentration, and the absorbance leveled off above 30 *μ*M. To assess the reaction kinetics, time-course of the excimer to monomer intensity ratio was measured with 30 *μ*M H_2_O_2_ (Figure S18). The intensity ratio gradually increased with increasing reaction time and then reached constant after 60 min. These observations indicate that the boronic acid groups were quantitatively converted into phenol moieties when H_2_O_2_ concentration is larger than 30 *μ*M, and the reaction of the boronic acid groups with H_2_O_2_ induced a conformational change in the copolymer from expanded to contracted state. It is calculated that the concentration of boronic acid unit in the measurement solutions of B-11 is ca. 3.5 *μ*M. (Polymer B-11 was synthesized from 76 mg (0.4 mmol) of** 1**, 28.4 mg of** 2**, and 11.4 mg of** 3**. Supposing that the monomer composition in the copolymer is the same as in the feed solution, the number of moles of boronic acid per unit weight of the copolymer is calculated to be 3.45 mmol g^−1^. Therefore, concentration of boronic acid unit in the measurement solutions containing 1 mg L^−1^ of B-11 is estimated to be 3.45 *μ*mol L^−1^.) However, the detection curve exhibited saturation at much higher H_2_O_2_ concentration. This means that H_2_O_2_ does not quantitatively react with the boronic acid unit. Probably the decomposition reaction of H_2_O_2_ into H_2_O and O_2_ is competing, and/or the reaction is relatively slow so that the reaction with boronic acid is uncompleted within 60 min at lower H_2_O_2_ concentrations.

The reaction of boronic acid with H_2_O_2_ to be converted into phenol was also confirmed by ^1^H NMR measurements. Firstly, phenylboronic acid was used as a model compound (Figure S20). In an alkaline D_2_O solution, phenylboronic acid showed signals in the range of 7.6–7.2 ppm. After the reaction with H_2_O_2_, it showed upfield shifts to 7.2–6.6 ppm that is assignable to the quantitative conversion to phenol. Then, copolymer B-11 was dissolved in alkaline D_2_O and NMR spectra were measured in the absence and presence of H_2_O_2_ (Figure S21). In the absence of H_2_O_2_, it showed broad peaks assignable to the boronic acid moiety at around 7.2 ppm. In the presence of excess amount of H_2_O_2_, the peaks disappeared and new peaks attributable to phenol moiety emerged in the range of 7.0–6.2 ppm.

**Figure 3:**
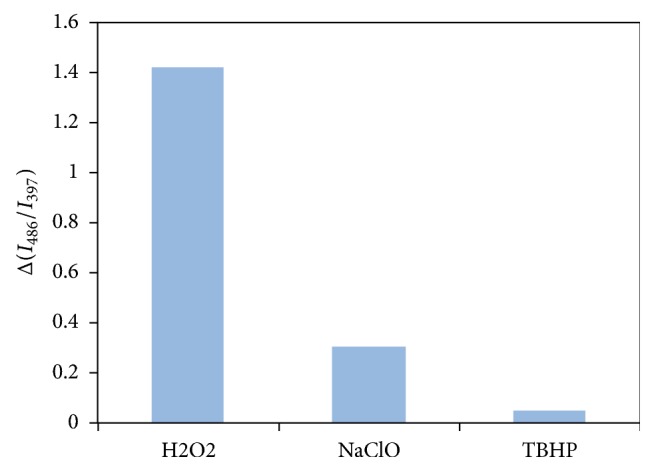
Comparison of the relative change in excimer (486 nm) to monomer (397 nm) emission intensity ratios against reactive oxygen species (30 *μ*M each) for aqueous solutions of B-11 buffered by 5 mM Na_2_CO_3_/5 mM NaHCO_3_ (pH 10.2).

We also examined detection selectivity against reactive oxygen species [26]. In these cases, measurements were conducted at pH 10.2 with sodium carbonate buffer instead of Good's buffer to avoid causing side reactions of reactive oxygen species with buffer. As the results shown in Figure 3, hypochlorite induced change in the ratio of excimer to monomer emission intensities that is much smaller than the case of H_2_O_2_. Also, t-butyl hydroperoxide (TBHP) showed nearly negligible response.

### 3.2. Effect of pH

**Figure 4:**
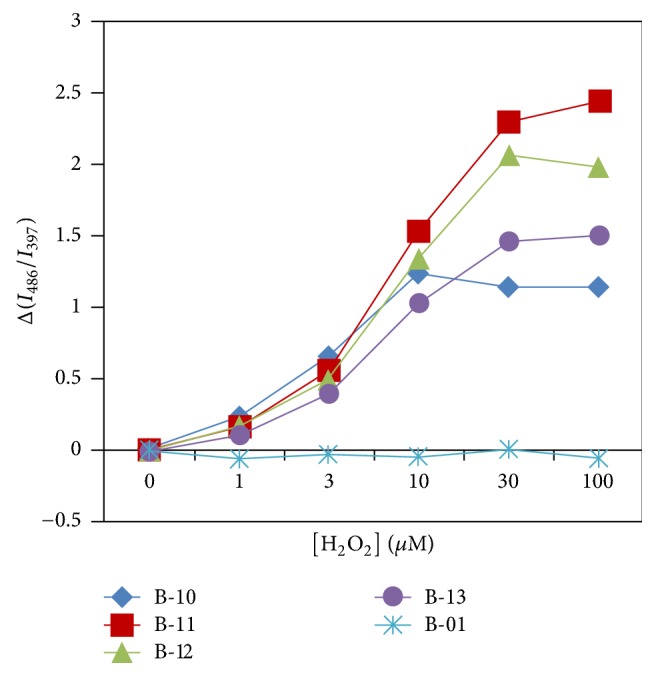
Ratio of excimer (486 nm) to monomer (397 nm) emission intensities for aqueous solutions of polymer B-11 containing (30 *μ*M) or not containing H_2_O_2_ as a function of pH at 25°C.

We further investigated the effect of pH on the responsiveness towards H_2_O_2_.  Figure 4 shows the pH dependency of the excimer to monomer intensity ratio in the absence and presence (30 *μ*M) of H_2_O_2_. At pH 7.4, there was no significant difference of intensity ratios in the absence and presence of H_2_O_2_. Unfortunately, further decrease in pH resulted in precipitation of the copolymer due to lack of boronate negative charges at acidic conditions. In the absence of H_2_O_2_, the relative intensity of excimer emission was almost constant with the increase in pH and then decreased when pH exceeded 10. The decrease in the excimer intensity may be due the extension of the polymer chain that was induced by the ionization of the boronic acid groups into negatively charged boronate groups [27] by which electrostatic repulsion was created within the polymer chain. This idea is supported by the study made by Chandar et al. on the conformations of a pyrene-labeled poly(acrylic acid) using fluorescence of the excimer/monomer emissions. They found that the polymer changed from coiled to extended form with increasing pH [28]. In the presence of 30 *μ*M of H_2_O_2_, the relative excimer intensity markedly increased with the increase in pH until 10.9, and it later decreased at pH 11.3. The increase in the excimer intensity suggests that the polymer chain bearing phenol moieties is contracted with the increase in pH. The phenol groups are expected to be partially dissociated at around pH 10 since p*K*_a_ values of monomeric phenols are typically around 10. We therefore speculated that the contraction was caused by the hydrogen bonding between the dissociated phenolate group and the undissociated phenol group [29]. This bonding should be stronger than the hydrogen bonding between phenols only since stronger electrostatic interaction occurs.

To confirm whether the above-mentioned interpretation is appropriate, effect of ionic strength on the response against H_2_O_2_ was evaluated (Figure S19). The response was largely suppressed in the presence of NaCl, which is attributable to disruption of the electrostatic interaction at the higher ionic strength. This result supports the view that the electrostatic attraction between phenol and phenolate is important for the response. The further increase in pH converts the undissociated phenol groups into dissociated phenolate groups, resulting in the creation of electrostatic repulsion between the negatively charged phenolate groups. Thus, the extension of the polymer chain is induced and the excimer emission intensity decreases. According to these results, it was found that the H_2_O_2_-responsive change in the fluorescence intensity ratio is maximal at pH 10.9.

### 3.3. Effect of Monomer Composition

**Figure 5:**
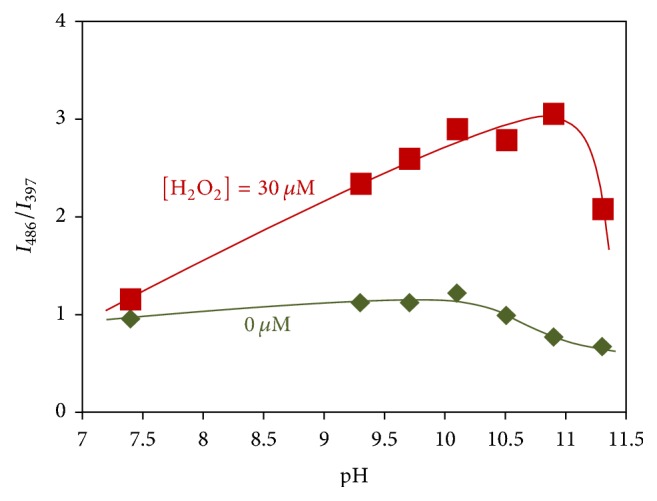
Relative change in excimer (486 nm) to monomer (397 nm) emission intensity ratios as a function of H_2_O_2_ concentrations for aqueous solutions of the polymers buffered by 10 mM CAPS (pH 10.9).

In order to evaluate the effect of boronic acid density on the polymer chain, copolymers having different monomer compositions (Table 1) were used. To compare responses of these polymers towards H_2_O_2_, the magnitude of increase in the excimer to monomer intensity ratio was plotted against H_2_O_2_ concentration, as shown in Figure 5. When compared with the response of polymer B-11, it is apparent that the decrease in the boronic acid content resulted in depression of the change in the excimer to monomer emission intensity ratios. Polymer B-12, which has a 1 : 2 molar ratio between the boronic acid and acrylamide units, exhibited a slightly diminished response. Moreover, polymer B-13 showed a further diminished response. In the case of polymer B-01, which does not have boronic acid unit, no virtual fluorescence response was induced. These observations clearly support the view that the existence of boronic acid group is indispensable for the occurrence of the responsiveness, and the reaction with H_2_O_2_ causes conformational alterations in the copolymer, resulting in the fluorescence spectral changes. Unexpectedly, polymer B-10, which has the highest boronic acid content, showed a much lower response as compared to polymer B-11. Polymer B-10 mainly consists of boronic acid units with a small content of pyrene units and no acrylamide unit. Since the boronic acid unit is sterically bulkier than acrylamide unit, the conformational flexibility of the polymer chain should be relatively restricted due to the steric hindrance between neighboring boronic acid moieties. This could be the reason for the suppression of response against H_2_O_2_.

## 4. Conclusions

**Scheme 3:**
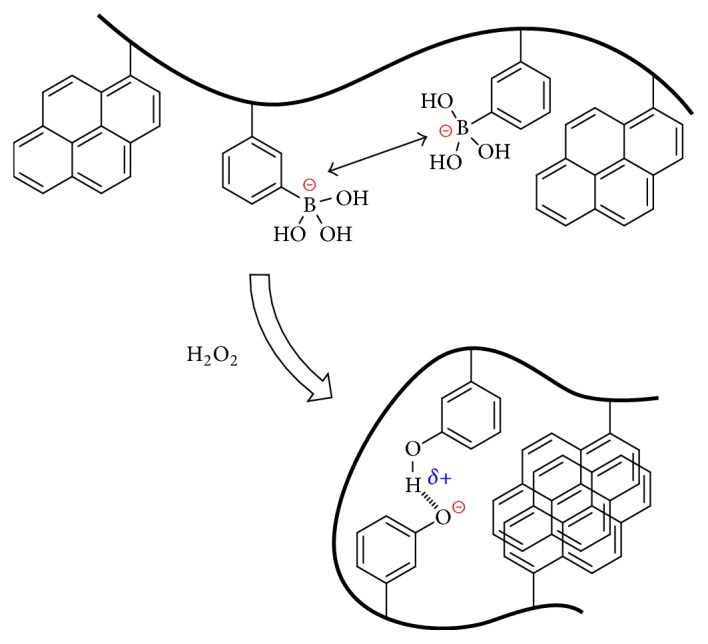
Conformational change of the fluorescent boronic acid polymer in response to H_2_O_2_.

In conclusion, we established that the copolymers containing boronic acid and pyrene units can be utilized for the fluorometric sensing of H_2_O_2_. The copolymer exists in a relatively extended conformation in the absence of H_2_O_2_, whereas the polymer chain is contracted by the reaction of the boronic acid moieties with H_2_O_2_. This conformational change induces aggregation of the originally isolated pyrene groups as illustrated in Scheme 3. As a result, relative intensity of excimer emission with respect to monomer emission increases with H_2_O_2_ concentration. Accordingly, the present methodology enables us to measure H_2_O_2_ by means of ratiometric fluorescence changes in the range of 0–30 *μ*M with the detection limit of 1 *μ*M.

## Supplementary Material

Figure S1: ^1^H NMR spectra of polymer **B-10** measured in DMSO-d_6_. Assignments are the same as those in Figure S2.
Figure S2: ^1^H NMR spectra of polymer **B-11** measured in DMSO-d_6_. Assignments are as follows: 9.701 ppm: amide (-CONH-), 8.211 and 8.045 ppm: aromatic (pyrene), 7.825-7.167 ppm: aromatic (boronic acid), 6.824 ppm: amide (-CONH_2_), 4.952 ppm: methylene in pyrene unit.
Figure S3: ^1^H NMR spectra of polymer **B-12** measured in DMSO-d_6_. Assignments are the same as those in Figure S2.
Figure S4: ^1^H NMR spectra of polymer **B-13** measured in DMSO-d_6_. Assignments are the same as those in Figure S2.
Figure S5: ^1^H NMR spectra of polymer **B-01** measured in DMSO-d_6_. Assignments are as follows: 8.5 – 8.0 ppm: aromatic (pyrene), 7.5 – 6.8 ppm: aromatic (boronic acid) and amide (-CONH_2_), 5.014 ppm: methylene in pyrene unit.
Table S1: Elemental analysis data.
Table S2: Estimated compositions of monomer units in copolymers.
Figure S6: Fluorescence spectra of polymer **B-10** (1 mg L^−1^) excited at 348 nm in an aqueous solution buffered at pH 10.9 (10 mM CAPS) at various H_2_O_2_ concentrations.
Figure S7: Fluorescence spectra of polymer **B-11** (1 mg L^−1^) excited at 348 nm in an aqueous solution buffered at pH 9.3 (10 mM CHES) at various H_2_O_2_ concentrations.
Figure S8: Fluorescence spectra of polymer **B-11** (1 mg L^−1^) excited at 348 nm in an aqueous solution buffered at pH 9.7 (10 mM CHES) at various H_2_O_2_ concentrations.
Figure S9: Fluorescence spectra of polymer **B-11** (1 mg L^−1^) excited at 348 nm in an aqueous solution buffered at pH 10.1 (10 mM CAPS) at various H_2_O_2_ concentrations.
Figure S10: Fluorescence spectra of polymer **B-11** (1 mg L^−1^) excited at 348 nm in an aqueous solution buffered at pH 10.5 (10 mM CAPS) at various H_2_O_2_ concentrations.
Figure S11: Fluorescence spectra of polymer **B-11** (1 mg L^−1^) excited at 348 nm in an aqueous solution buffered at pH 10.9 (10 mM CAPS) at various H_2_O_2_ concentrations.
Figure S12: Fluorescence spectra of polymer **B-11** (1 mg L^−1^) excited at 348 nm in an aqueous solution buffered at pH 11.3 (10 mM CAPS) at various H_2_O_2_ concentrations.
Figure S13: Fluorescence spectra of polymer **B-12** (1 mg L^−1^) excited at 348 nm in an aqueous solution buffered at pH 10.9 (10 mM CAPS) at various H_2_O_2_ concentrations.
Figure S14: Fluorescence spectra of polymer **B-13** (1 mg L^−1^) excited at 348 nm in an aqueous solution buffered at pH 10.9 (10 mM CAPS) at various H_2_O_2_ concentrations.
Figure S15: Fluorescence spectra of polymer **B-01** (1 mg L^−1^) excited at 348 nm in an aqueous solution buffered at pH 10.9 (10 mM CAPS) at various H_2_O_2_ concentrations.
Figure S16: Fluorescence excitation spectra of polymer **B-11** (1 mg L^−1^) observed at 397 nm and 486 nm in an aqueous solution buffered at pH 10.9 (10 mM CAPS) in the absence of H_2_O_2_. The spectra are normalized at 348 nm.
Figure S17: Absorption spectra of polymer **B-11** (1 mg L^−1^) in an aqueous solution buffered at pH 10.9 (10 mM CAPS) at various H_2_O_2_ concentrations; inset shows the relationship between absorbance at 290 nm and H_2_O_2_ concentration.
Figure S18: Time-course of excimer to monomer emission intensity ratio for an aqueous solution of polymer **B-11** (1 mg L^−1^) buffered at pH 10.9 (10 mM CAPS) containing 30 μM H_2_O_2_.
Figure S19: Ratio of excimer to monomer emission intensities as a function of H_2_O_2_ concentrations for aqueous solutions of polymer **B-11** (1 mg L^−1^) buffered at pH 10.9 (10 mM CAPS) at various concentrations of NaCl.
Figure S20: ^1^H NMR spectra of (a) 5 mM phenylboronic acid, and (b) 5 mM phenylboronic acid + 10 mM H_2_O_2_ measured in D_2_O containing 50 mM Na_2_CO_3_.
Figure S21: ^1^H NMR spectra of (a) B-11, and (b) B-11 + 20 mM H_2_O_2_ measured in D_2_O containing 50 mM Na_2_CO_3_. Polymer concentration was 2 g L^−1^ ([boronic acid] = 7 mM).

## Figures and Tables

**Table 1:** Monomer compositions of copolymers^a^.

Polymer	Monomer (*μ*mol)		
	**1**	**2**	**3**
B-10	800	0	40
B-11	400	400	40
B-12	300	600	45
B-13	200	600	40
B-01	0	800	40

^a^Solvent: DMSO 2 mL + H_2_O 0.1 mL.
