# Supplementary material for: Ratiometric Sensing of Hydrogen Peroxide Utilizing Conformational Change in Fluorescent Boronic Acid Polymers
Source: J Anal Methods Chem. 2017 Sep 28;2017:7829438. doi: 10.1155/2017/7829438 (PMC5637826; doi:10.1155/2017/7829438)
Supplement: Supplementary file 1 — Figure S1: 1H NMR spectra of polymer B-10 measured in DMSO-d6. Assignments are the same as those in Figure S2. Figure S2: 1H NMR spectra of polymer B-11 measured in DMSO-d6. Assignments are as follows: 9.701 ppm: amide (-CONH-), 8.211 and 8.045 ppm: aromatic (pyrene), 7.825-7.167 ppm: aromatic (boronic acid), 6.824 ppm: amide (-CONH2), 4.952 ppm: methylene in pyrene unit. Figure S3: 1H NMR spectra of polymer B-12 measured in DMSO-d6. Assignments are the same as those in Figure S2. Figure S4: 1H NMR spectra of polymer B-13 measured in DMSO-d6. Assignments are the same as those in Figure S2. Figure S5: 1H NMR spectra of polymer B-01 measured in DMSO-d6. Assignments are as follows: 8.5 – 8.0 ppm: aromatic (pyrene), 7.5 – 6.8 ppm: aromatic (boronic acid) and amide (-CONH2), 5.014 ppm: methylene in pyrene unit. Table S1: Elemental analysis data. Table S2: Estimated compositions of monomer units in copolymers. Figure S6: Fluorescence spectra of polymer B-10 (1 mg L−1) excited at 348 nm in an aqueous solution buffered at pH 10.9 (10 mM CAPS) at various H2O2 concentrations. Figure S7: Fluorescence spectra of polymer B-11 (1 mg L−1) excited at 348 nm in an aqueous solution buffered at pH 9.3 (10 mM CHES) at various H2O2 concentrations. Figure S8: Fluorescence spectra of polymer B-11 (1 mg L−1) excited at 348 nm in an aqueous solution buffered at pH 9.7 (10 mM CHES) at various H2O2 concentrations. Figure S9: Fluorescence spectra of polymer B-11 (1 mg L−1) excited at 348 nm in an aqueous solution buffered at pH 10.1 (10 mM CAPS) at various H2O2 concentrations. Figure S10: Fluorescence spectra of polymer B-11 (1 mg L−1) excited at 348 nm in an aqueous solution buffered at pH 10.5 (10 mM CAPS) at various H2O2 concentrations. Figure S11: Fluorescence spectra of polymer B-11 (1 mg L−1) excited at 348 nm in an aqueous solution buffered at pH 10.9 (10 mM CAPS) at various H2O2 concentrations. Figure S12: Fluorescence spectra of polymer B-11 (1 mg L−1) excited at 348 nm in an aqueous [file 7829438.f1.docx]

Supplementary Materials

| **Ratiometric Sensing of Hydrogen Peroxide utilizing Conformational Change in Fluorescent Boronic Acid Polymers**  **Kan Takeshima,^a^ Kanako Mizuno,^a^ Hitoshi Nakahashi,^a^ Horoshi Aoki,^b^ and Yasumasa Kanekiyo*^a^**  **^a^** Department of Biotechnology and Environmental Chemistry, Kitami Institute of Technology,  165 Koen-cho, Kitami, Hokkaido 090-8507, Japan.  **^b^** National Institute of Advanced Industrial Science and Technology (AIST), 16-1 Onogawa, Tsukuba, Ibaraki 305-8569, Japan. |
| --- |


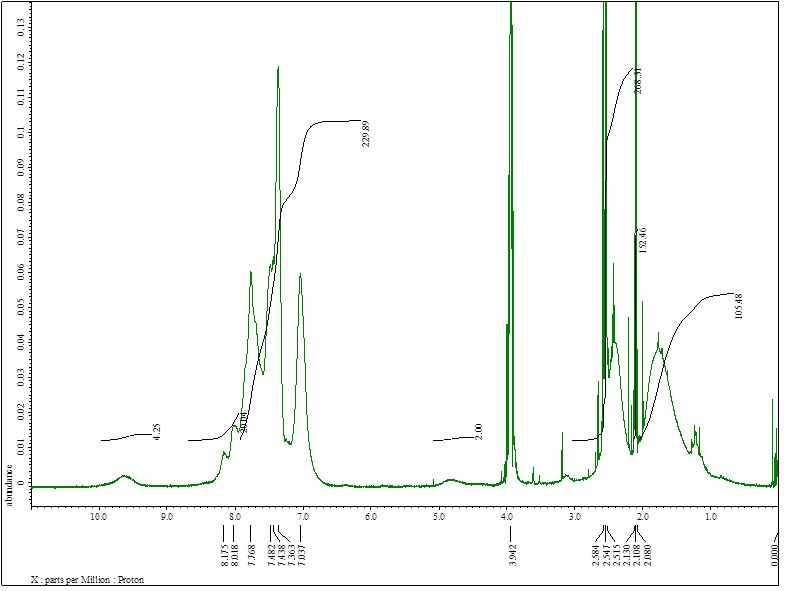


**Figure S1** ^1^H NMR spectra of polymer **B-10** measured in DMSO-d_6_. Assignments are the same as those in Figure S2.


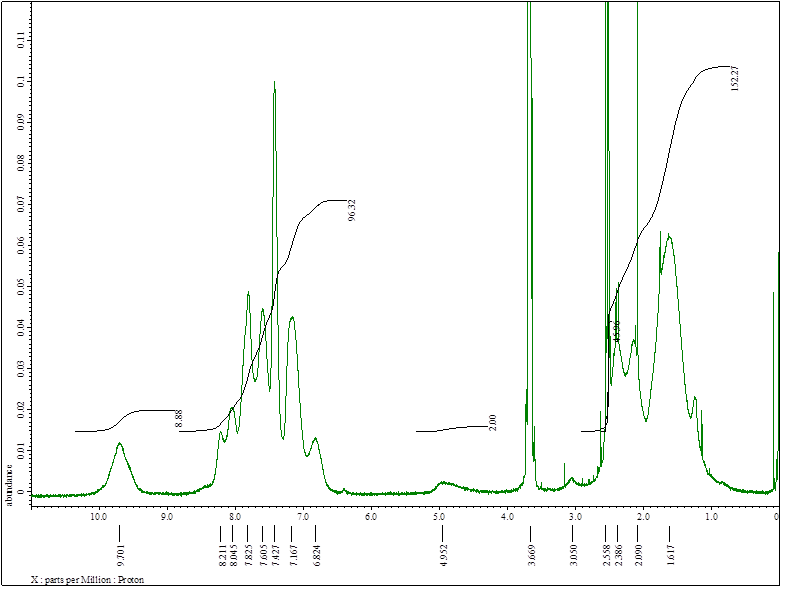


**Figure S2** ^1^H NMR spectra of polymer **B-11** measured in DMSO-d_6_. Assignments are as follows: 9.701 ppm: amide (-CONH-), 8.211 and 8.045 ppm: aromatic (pyrene), 7.825-7.167 ppm: aromatic (boronic acid), 6.824 ppm: amide (-CONH_2_), 4.952 ppm: methylene in pyrene unit.


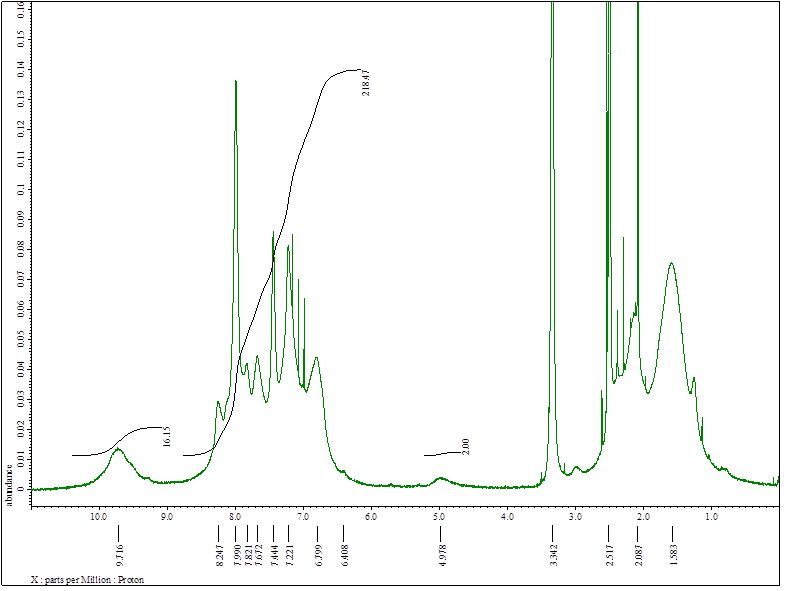


**Figure S3** ^1^H NMR spectra of polymer **B-12** measured in DMSO-d_6_. Assignments are the same as those in Figure S2.


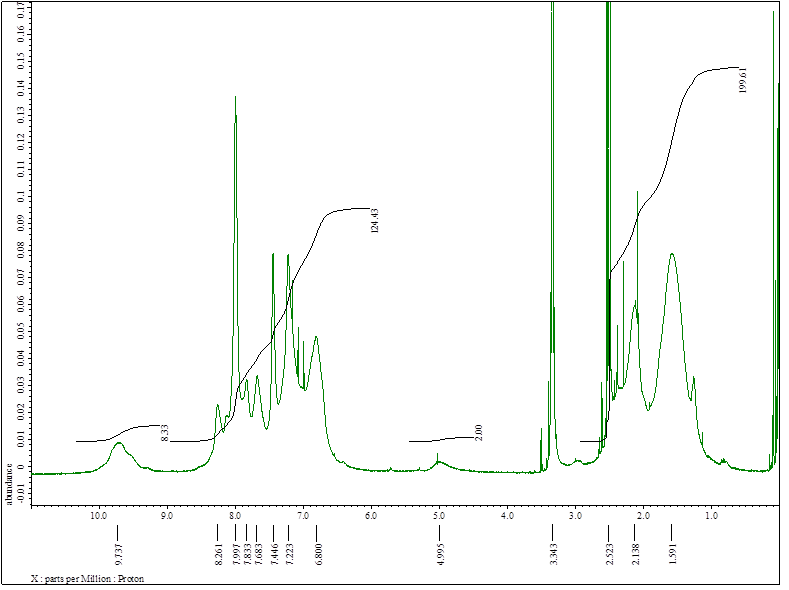


**Figure S4** ^1^H NMR spectra of polymer **B-13** measured in DMSO-d_6_. Assignments are the same as those in Figure S2.


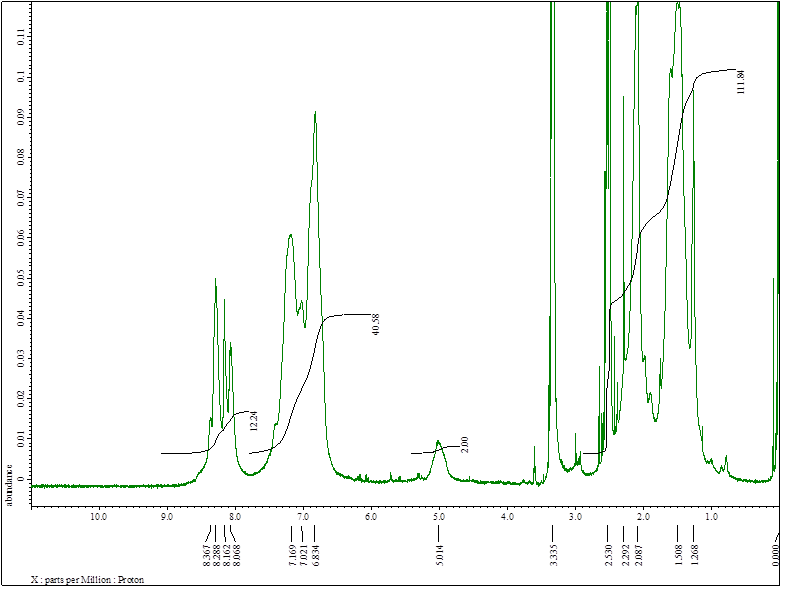


**Figure S5** ^1^H NMR spectra of polymer **B-01** measured in DMSO-d_6_. Assignments are as follows: 8.5 – 8.0 ppm: aromatic (pyrene), 7.5 – 6.8 ppm: aromatic (boronic acid) and amide (-CONH_2_), 5.014 ppm: methylene in pyrene unit.

**Table S1** Elemental analysis data

| Sample | Elemental data / wt% | | |  |  |
| --- | --- | --- | --- | --- | --- |
|  | C | H | N | C / N (obs.) | C / N (calc.) |
| **B-10** | 55.16 | 5.84 | 6.53 | 8.45 | 8.15 |
| **B-11** | 52.98 | 6.14 | 9.33 | 5.68 | 5.70 |
| **B-12** | 51.10 | 6.36 | 10.60 | 4.82 | 4.90 |
| **B-01** | 50.37 | 7.32 | 15.67 | 3.25 | 3.21 |

**Table S2** Estimated compositions of monomer units in copolymers

| Sample | Molar ratio / % | | |
| --- | --- | --- | --- |
|  | **1** | **2** | **3** |
| **B-10**^*^ | 92.2 | 0 | 7.8 |
| **B-11**^*^ | 46.9 | 48.3 | 4.8 |
| **B-12**^*^ | 30.3 | 65.0 | 4.8 |
| **B-01**^*^ | 0 | 96.6 | 4.4 |

^*^Molar ratio of **3** is supposed to be the same as in the feed solutions


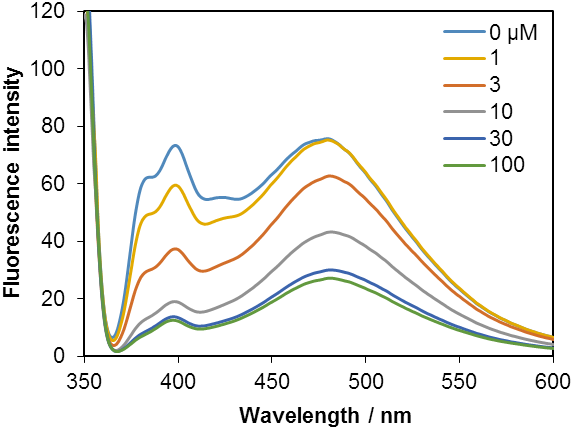


**Figure S6** Fluorescence spectra of polymer **B-10** (1 mg L^-1^) excited at 348 nm in an aqueous solution buffered at pH 10.9 (10 mM CAPS) at various H_2_O_2_ concentrations.


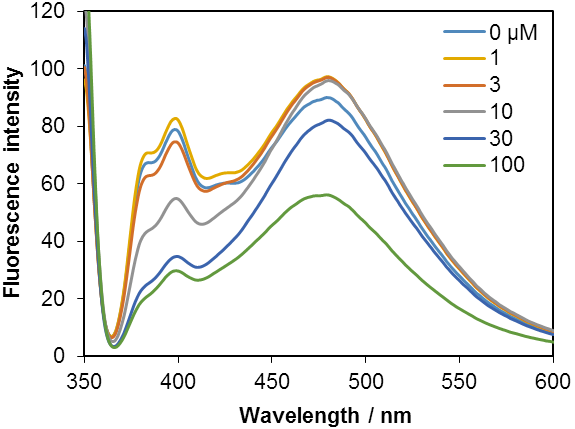


**Figure S7** Fluorescence spectra of polymer **B-11** (1 mg L^-1^) excited at 348 nm in an aqueous solution buffered at pH 9.3 (10 mM CHES) at various H_2_O_2_ concentrations.


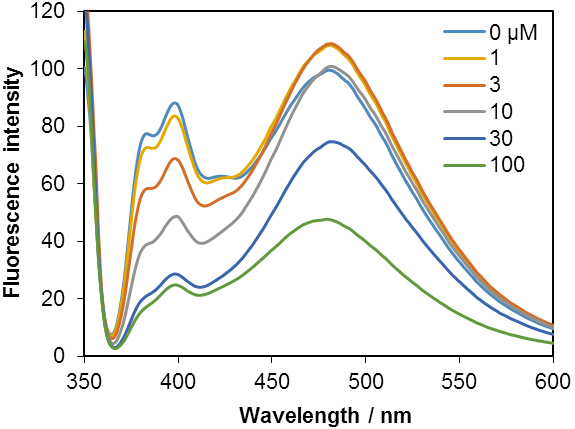


**Figure S8** Fluorescence spectra of polymer **B-11** (1 mg L^-1^) excited at 348 nm in an aqueous solution buffered at pH 9.7 (10 mM CHES) at various H_2_O_2_ concentrations.


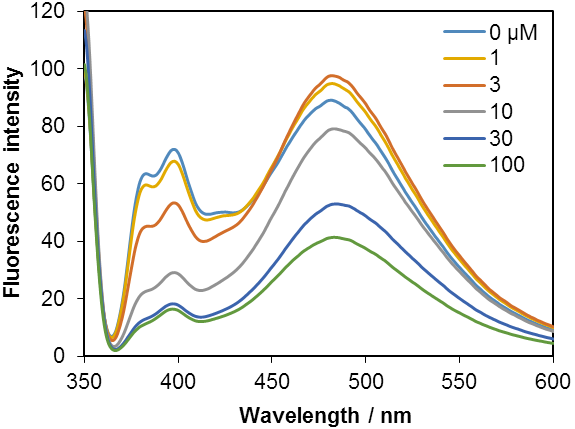


**Figure S9** Fluorescence spectra of polymer **B-11** (1 mg L^-1^) excited at 348 nm in an aqueous solution buffered at pH 10.1 (10 mM CAPS) at various H_2_O_2_ concentrations.


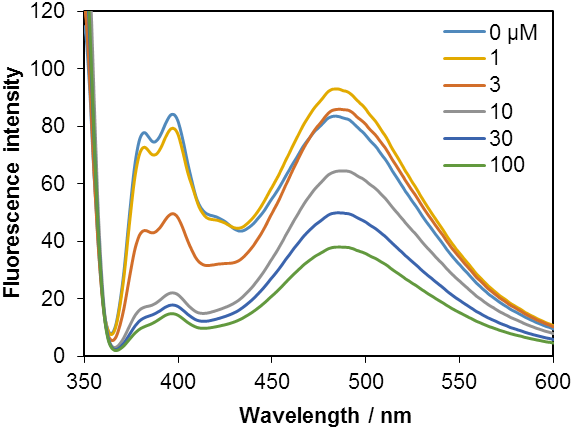


**Figure S10** Fluorescence spectra of polymer **B-11** (1 mg L^-1^) excited at 348 nm in an aqueous solution buffered at pH 10.5 (10 mM CAPS) at various H_2_O_2_ concentrations.


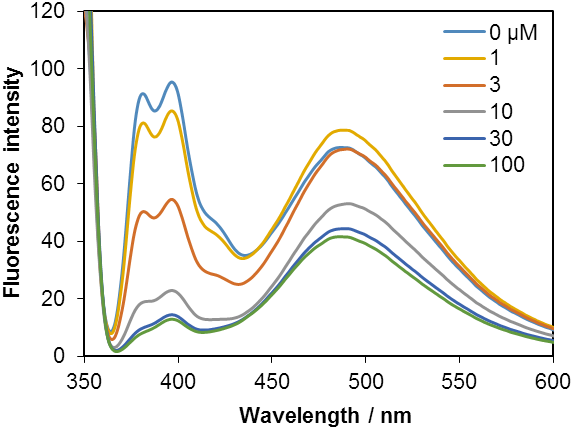


**Figure S11** Fluorescence spectra of polymer **B-11** (1 mg L^-1^) excited at 348 nm in an aqueous solution buffered at pH 10.9 (10 mM CAPS) at various H_2_O_2_ concentrations.


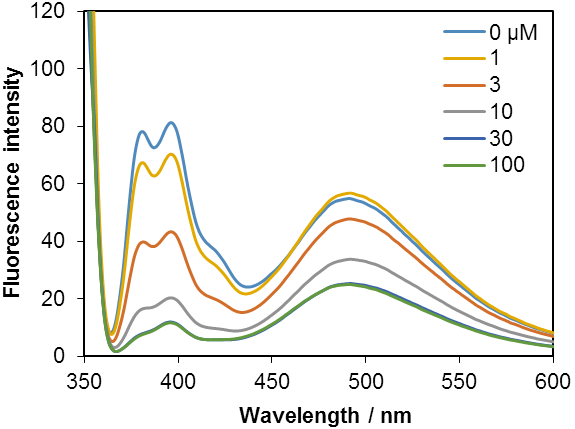


**Figure S12** Fluorescence spectra of polymer **B-11** (1 mg L^-1^) excited at 348 nm in an aqueous solution buffered at pH 11.3 (10 mM CAPS) at various H_2_O_2_ concentrations.


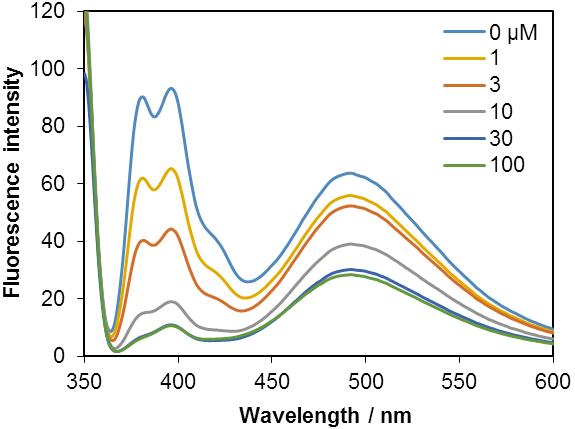


**Figure S13** Fluorescence spectra of polymer **B-12** (1 mg L^-1^) excited at 348 nm in an aqueous solution buffered at pH 10.9 (10 mM CAPS) at various H_2_O_2_ concentrations.


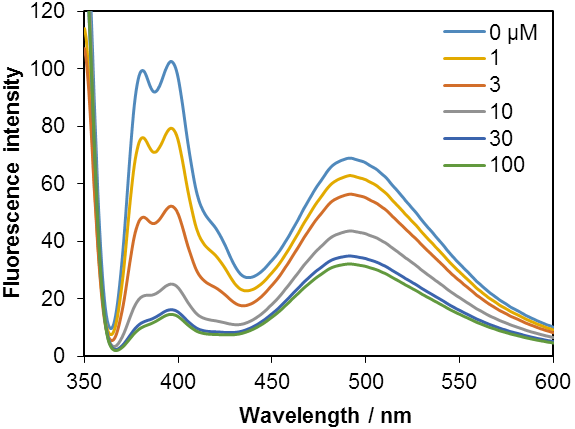


**Figure S14** Fluorescence spectra of polymer **B-13** (1 mg L^-1^) excited at 348 nm in an aqueous solution buffered at pH 10.9 (10 mM CAPS) at various H_2_O_2_ concentrations.


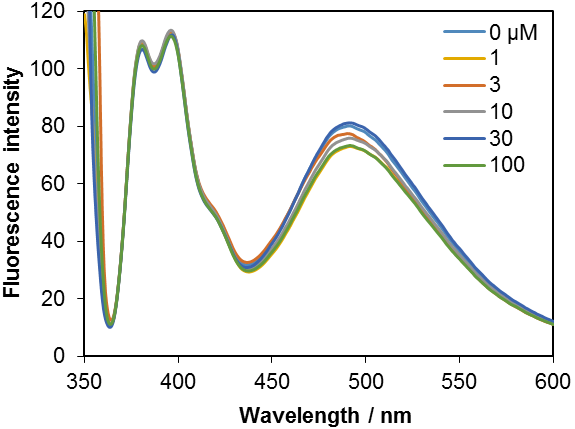


**Figure S15** Fluorescence spectra of polymer **B-01** (1 mg L^-1^) excited at 348 nm in an aqueous solution buffered at pH 10.9 (10 mM CAPS) at various H_2_O_2_ concentrations.


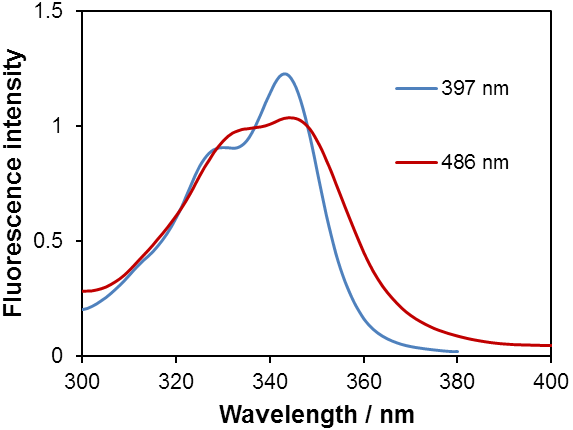


**Figure S16** Fluorescence excitation spectra of polymer **B-11** (1 mg L^-1^) observed at 397 nm and 486 nm in an aqueous solution buffered at pH 10.9 (10 mM CAPS) in the absence of H_2_O_2_. The spectra are normalized at 348 nm.


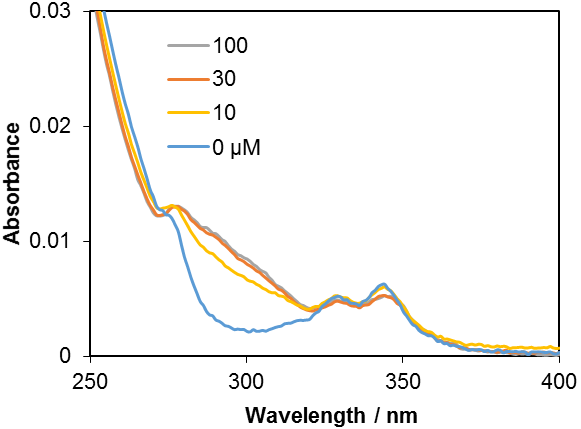

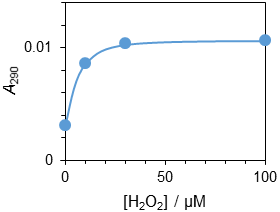


**Figure S17** Absorption spectra of polymer **B-11** (1 mg L^-1^) in an aqueous solution buffered at pH 10.9 (10 mM CAPS) at various H_2_O_2_ concentrations; inset shows the relationship between absorbance at 290 nm and H_2_O_2_ concentration.


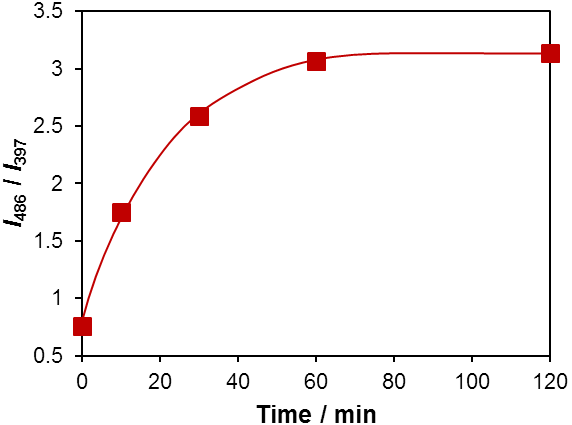


**Figure S18** Time-course of excimer to monomer emission intensity ratio for an aqueous solution of polymer **B-11** (1 mg L^-1^) buffered at pH 10.9 (10 mM CAPS) containing 30 μM H_2_O_2_.


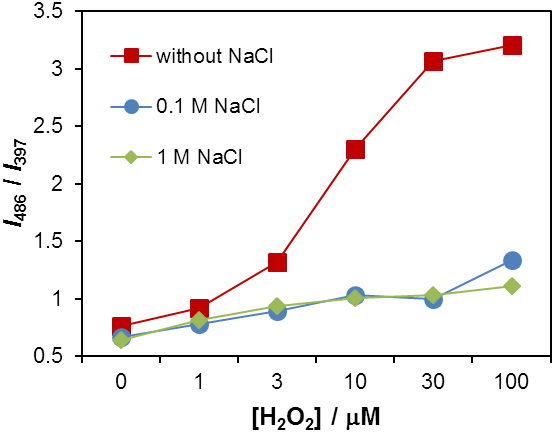


**Figure S19** Ratio of excimer to monomer emission intensities as a function of H_2_O_2_ concentrations for aqueous solutions of polymer **B-11** (1 mg L^-1^) buffered at pH 10.9 (10 mM CAPS) at various concentrations of NaCl.

ppm

ppm

a

b

**Figure S20** ^1^H NMR spectra of (a) 5 mM phenylboronic acid, and (b) 5 mM phenylboronic acid + 10 mM H_2_O_2_ measured in D_2_O containing 50 mM Na_2_CO_3_.


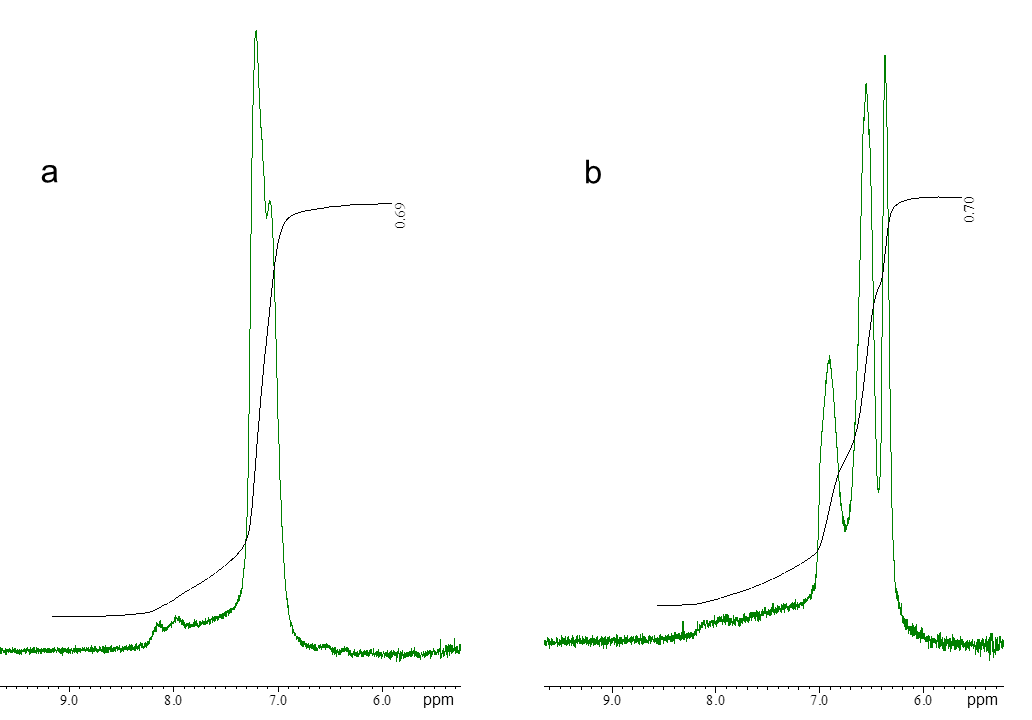


**Figure S21** ^1^H NMR spectra of (a) B-11, and (b) B-11 + 20 mM H_2_O_2_ measured in D_2_O containing 50 mM Na_2_CO_3_. Polymer concentration was 2 g L^-1^ ([boronic acid] = 7 mM).
